# Transcatheter Repair of Ventricular Septal Defect Post Myocardial Infarction at a Community Hospital: A Case Study

**DOI:** 10.7759/cureus.87246

**Published:** 2025-07-03

**Authors:** Gurpreet Singh, Rajveer s Sangera, Vishal Patel, Shea D Aiken

**Affiliations:** 1 Medicine, Providence Santa Rosa Memorial Hospital, Santa Rosa, USA; 2 Interventional Cardiology, Providence Santa Rosa Memorial Hospital, Santa Rosa, USA; 3 Anesthesiology, Providence Santa Rosa Memorial Hospital, Santa Rosa, USA

**Keywords:** acute myocardial infarction, ischemic vsd repair, post myocardial infarction vsd, transcatheter intervention, ventricular septal defect (vsd)

## Abstract

Ventricular septal defect (VSD) is a rare complication following a myocardial infarction (MI), with a very high mortality rate if left untreated. Traditionally, surgical repair has been used to treat VSDs, but even in those cases, it has poor outcomes. This case shows a middle-aged woman who suffered an MI complicated by a VSD successfully treated with a transcatheter approach to repair the defect. The procedure was performed at a non-teaching community hospital with a small cardiology team. This case highlights the emerging use and success of non-invasive repair in post MI-associated VSD.

## Introduction

Ventricular septal defect (VSD) is a common congenital heart defect that can also occur as a complication of myocardial infarction (MI), occurring in approximately 1-2% of patients with acute MI. Following the adoption of reperfusion therapies as the standard treatment for acute MI, the incidence has decreased to a range of 0.17-0.31% [1]. VSDs commonly present in a bimodal manner, with a higher incidence in the initial 24 hours after acute MI, and then again three to five days later. In such cases, the defect can create a shunt between the left and right ventricles, leading to significant hemodynamic changes and potential heart failure [2]. Traditionally, surgical repair has been the standard approach for treating ventricular septal defects, but this procedure can be associated with significant morbidity and mortality, especially in high-risk patients [3]. The Amplatzer post-infarction ventricular septal defect occluder, a minimally invasive percutaneous device, represents the only FDA-approved alternative to surgical closure for patients who are not suitable candidates for the traditional open-heart procedure [4]. Percutaneous transcatheter closures are typically performed at tertiary care centers; this case highlights the successful utilization of this treatment at a community hospital, indicating the broader applicability of this approach in varied clinical settings.

## Case presentation

**Figure 1:**
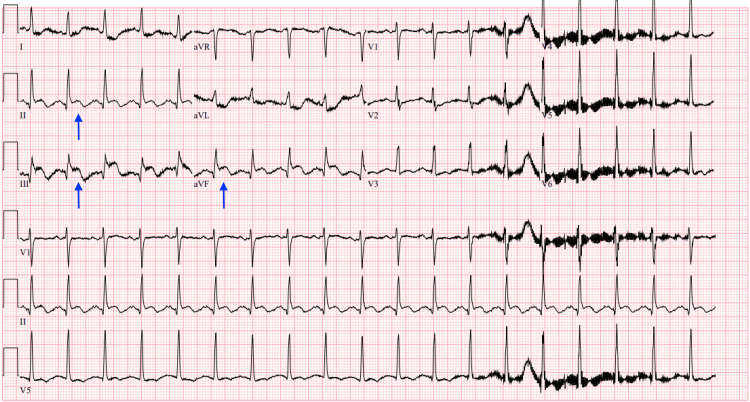
Initial EKG showing inferior STEMI with arrows showing ST elevations in leads II, III, and aVF STEMI: ST-elevation myocardial infarction

We present a case of a 73-year-old female who has a history of coronary artery disease, obesity, and prediabetes who came in with chest pain described as substernal chest pressure and tightness. Her symptoms started seven hours prior to her arrival at the hospital. EKG revealed ST elevations in the inferior leads, consistent with an acute MI (Figure 1).

**Figure 2:**
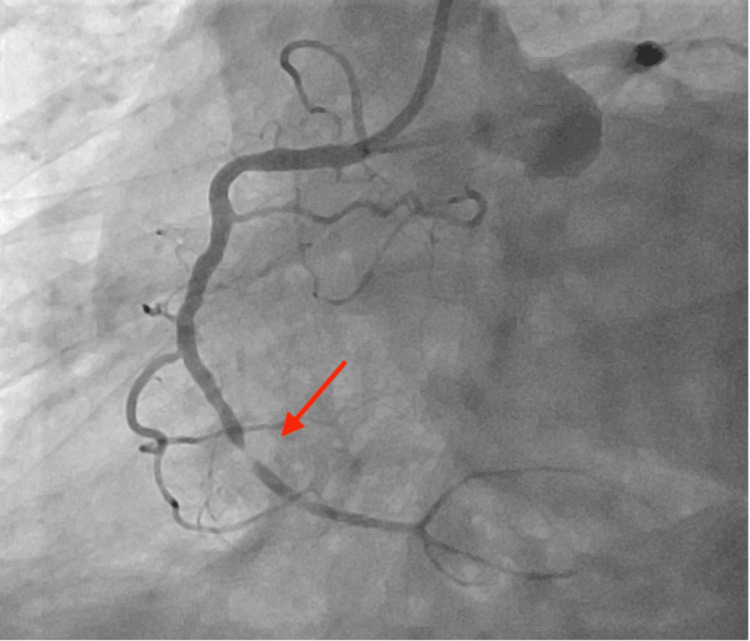
Right coronary artery occlusion prior to cardiac intervention, with the arrow showing the area of stenosis

**Figure 3:**
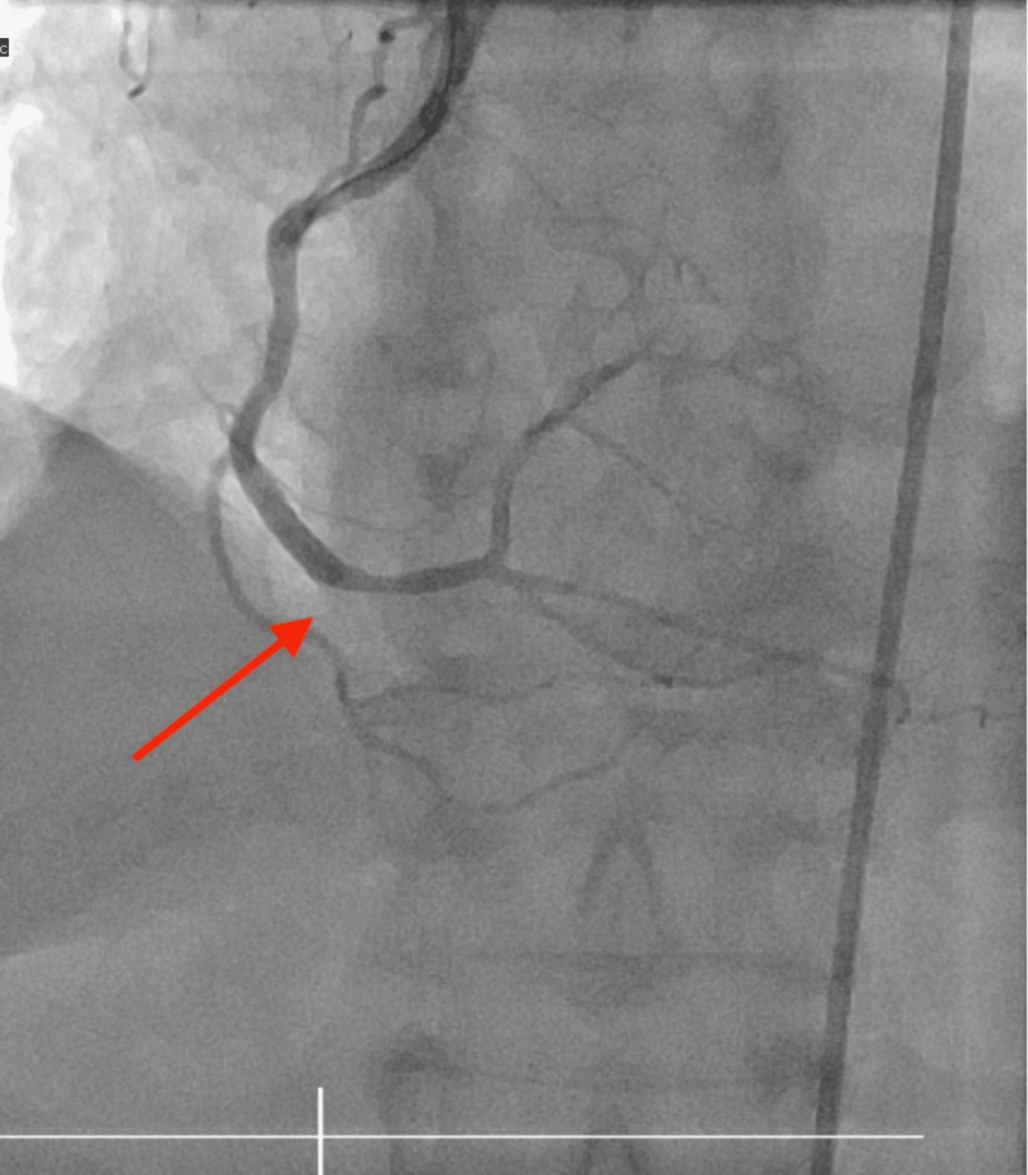
Post cardiac stenting of the right coronary artery, with the arrow showing the area of revascularization of the vessel

Labs were significant for initial troponin I of 4,575 ng/L. The patient underwent emergent cardiac catheterization and was found to have an occlusion of the right coronary artery (Figure 2). Percutaneous coronary intervention with drug-eluting stent placement was performed. Severe distal right coronary artery 99% stenosis was treated with percutaneous cardiac intervention with one drug-eluting stent 3.0 x 18 mm post-dilated to 3.5 mm (Figure 3), and the remainder of the coronaries were nonobstructive.

**Figure 4:**
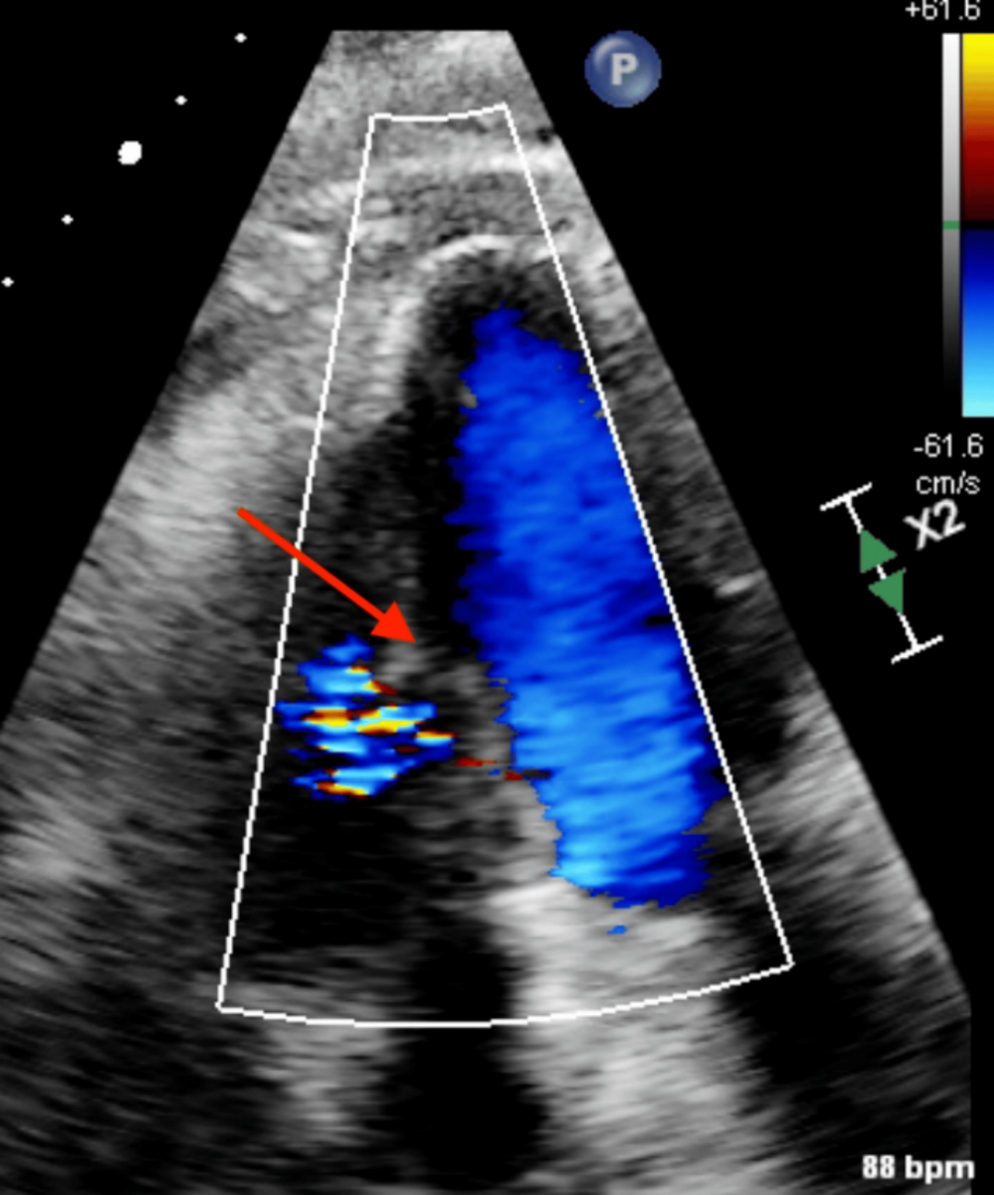
Echocardiogram done day one after the right coronary artery stent showing the ventricular septal defect

Post cardiac catheterization transthoracic echocardiogram on day one revealed normal left ventricular wall thickness and cavity size, and a muscular ventricular septal defect was identified, measuring 0.72 cm with left-to-right shunting (Figure 4).

**Figure 5:**
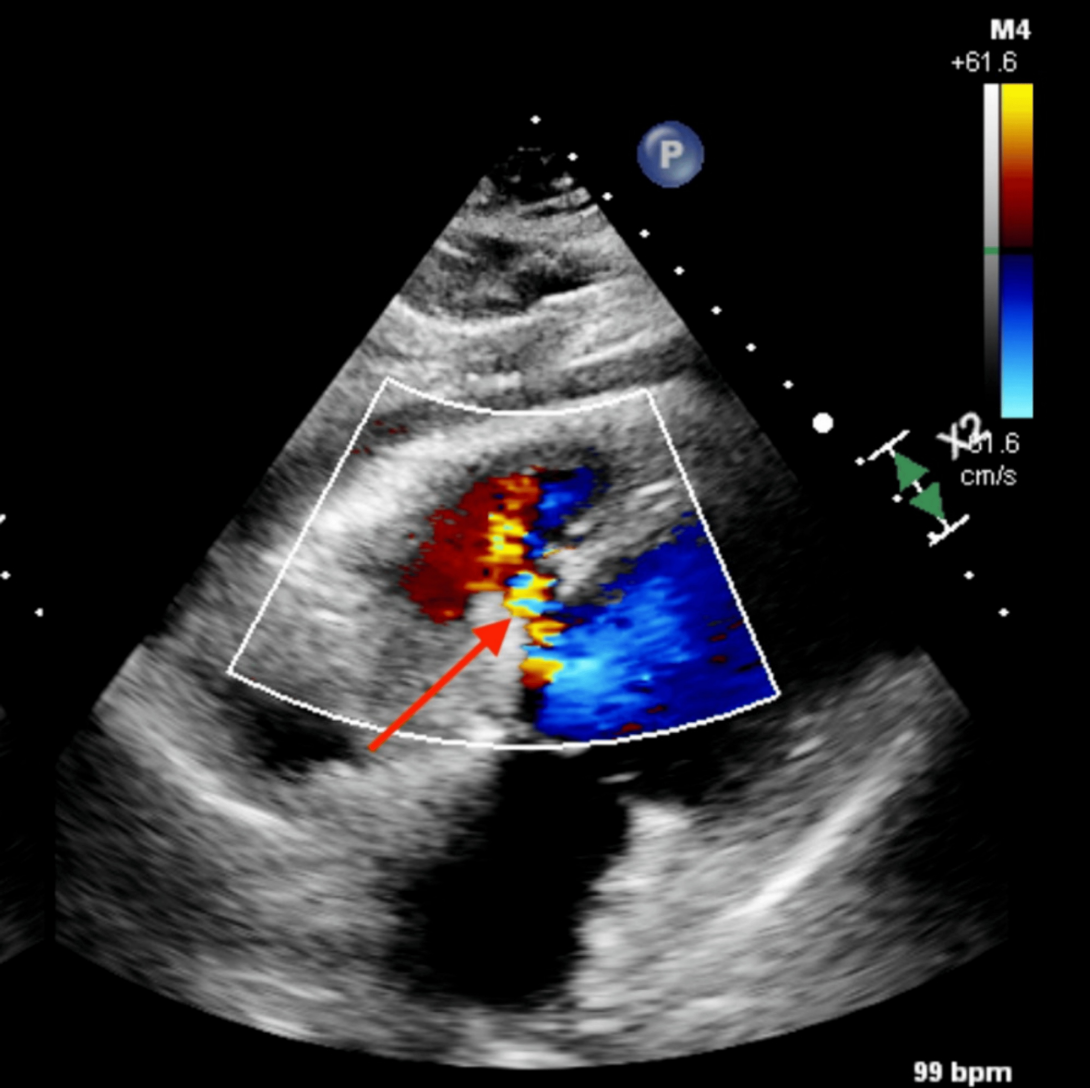
Large ventricular septal defect five days post myocardial infarction

Cardiac catheterization revealed a Qp/Qs of 2.33/1, severely elevated pulmonary pressures, mean PA of 38 mmHg, pulmonary capillary wedge pressure of 22 mmHg, and normal Fick cardiac output/index. On hospital day five, a transthoracic echocardiogram was repeated to check its progression prior to the procedure, which revealed a large ventricular septal defect measuring 1.25 cm with significant left-to-right shunting (Figure 5).

**Figure 6:**
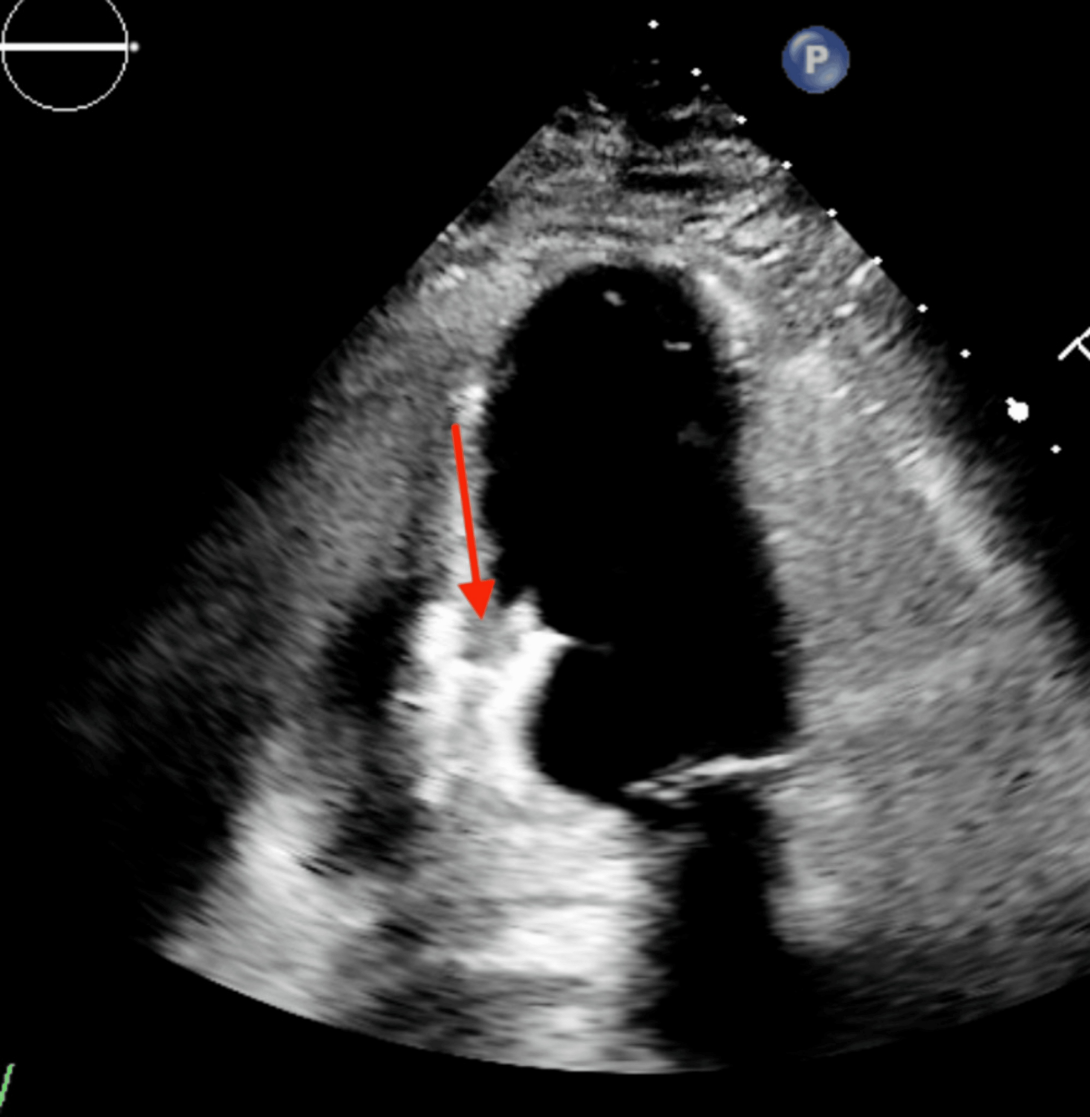
Echocardiogram after placement of the ventricular septal defect closure device implantation

Given the large size of the defect and the patient's poor comorbidities, the cardiothoracic surgery team felt that surgical repair would carry a very high mortality risk. After a multidisciplinary discussion, the patient was referred for percutaneous transcatheter closure of the ventricular septal defect. Through percutaneous access from the right common femoral vein and right internal jugular vein, a 20 mm Amplatzer ventricular septal defect occluder was deployed without incident across the ventricular septal defect. Subsequent left ventriculogram showed significant resolution of the defect with only mild shunting. This was also confirmed on a transesophageal echocardiogram. The patient tolerated the procedure well with no complications. The procedure was performed under fluoroscopy and completed in 66 minutes. Post-deployment transthoracic echocardiogram revealed the device in good position with a trivial residual shunt across the device (Figure 6).

The patient tolerated the procedure well with no complications. She was monitored post procedure in the intensive care unit for 24 hours and discharged home the following day. The patient is doing well clinically on her follow-up appointments after three months but has yet to complete a follow-up echocardiogram to completely quantify the results.

## Discussion

MI can lead to the development of a ventricular septal defect through a few key pathophysiological mechanisms. The infarcted myocardial tissue loses its contractile function, resulting in decreased ventricular wall motion and an inability to adequately seal the interventricular septum. Additionally, the increased wall stress and pressure in the left ventricle can lead to the disruption of the weakened septal tissue and the formation of the defect [5]. Unpredictable hemodynamic deterioration can rapidly develop in 80% of the patients, and mortality with medical therapy alone exceeds 90% [6]. Initial medical therapy for post-infarct VSR should center on hemodynamic stabilization and afterload reduction with vasodilators if possible. Transthoracic and Doppler echocardiography are essential to diagnosing the presence, size, and hemodynamic impact of a ventricular septal defect and helping establish the diagnosis while excluding other etiologies of hemodynamic instability [7].

In this case, the patient underwent successful transcatheter closure of the ventricular septal defect. The defect was successfully occluded using a percutaneously delivered occluder device, which was carefully positioned to minimize residual shunting. This minimally invasive approach offers a distinct advantage over traditional open-heart surgery, avoiding the associated risks of cardiopulmonary bypass, extensive surgical incisions, and prolonged post-operative recovery, which are particularly relevant in patients with compromised cardiac function and multiple comorbidities. The reported mortality in the United States, if surgically repaired within one week, is 54.1%, which constitutes one of the greatest mortality rates in the modern era of cardiac surgery [8]. The Society of Thoracic Surgeons National Database analysis of 2,876 patients with ventricular septal rupture revealed an overall in-hospital or 30-day mortality rate of 42.9%, which represents the highest mortality among all cardiac surgical procedures. Notably, a sharp decrease in mortality was observed with delayed surgical repair, with a 54.1% mortality rate for repairs conducted within seven days of MI, compared to a significantly lower 18.4% mortality rate for repairs performed after seven days from the initial infarction [8]. The difference in mortality rates can be explained by initial instability during the early remodeling period, which is why delaying surgery tends to have better outcomes if the patient survives past the late remodeling period (>72 hours) [9,10]. To address these concerns, percutaneous transcatheter closure of post MI ventricular septal defects has emerged as an appealing alternative to surgical repair, offering a minimally invasive approach to reducing the shunt and improving hemodynamic function.

In a review of 13 case series encompassing a total of 273 patients treated with transcatheter closure, the overall procedure success rate is 89%, and the 30-day or in-hospital mortality is 32%. Great heterogeneity concerning the timing of the interventional approach was observed [11]. Despite the advancement in surgical and percutaneous therapies, post MI ventricular septal defect repair is still associated with significant mortality and morbidity [12]. Although direct comparisons between surgical and interventional closures are limited by the non-randomized design and selection bias inherent in case series, percutaneous VSD closure presents a valuable opportunity to circumvent or postpone surgical intervention, potentially offering critical therapy to high-risk patients with ventricular septal defects.

Oversizing the disc may improve procedure success by accounting for defect enlargement due to tissue necrosis. Challenges to TSC include inferior defects due to a lack of a circumferential septal rim, basal defects due to the proximity to the tricuspid valvular apparatus, serpiginous defects due to complicated morphology, and closure soon following infarction due to tissue instability. Consensus opinion regarding optimal timing for closure is open to interpretation in the absence of high-level evidence. Arrhythmia represents the most frequently observed complication following transcatheter device insertion for VSDs. Embolism and endocarditis are less commonly encountered [13].

The case presented here highlights the successful application of this approach in a non-teaching community hospital setting. The length of the procedure was less than two hours, and the patient avoided cardiopulmonary bypass and had a short recovery time. The interventional cardiology department in the hospital consists of a total of three interventional cardiologists, all board-certified and experienced in structural heart disease interventions. Cardiothoracic surgery support was available if needed. It was the first time this procedure was performed in the hospital, and it showed that transcatheter closure could be expanded to non-academic centers with proper training and teamwork.

## Conclusions

This case highlights the successful use of transcatheter repair for a ventricular septal defect following MI at a community hospital. The patient had good short-term outcomes. As such, transcatheter repair may offer a valuable option for patients who are poor candidates for open-heart surgery.
